# Overcoming Challenges of Incorporation of Biobased Dibutyl Itaconate in (Meth)acrylic Waterborne Polymers

**DOI:** 10.1021/acs.biomac.4c00739

**Published:** 2024-07-31

**Authors:** Jyoti Gupta, Radmila Tomovska, Miren Aguirre

**Affiliations:** † POLYMAT, Kimika Aplikatua Saila, Kimika Fakultatea, University of the Basque Country UPV-EHU, Joxe Mari Korta Zentroa, Tolosa Hiribidea 72, 20018 Donostia-San Sebastián, Spain; ‡ IKERBASQUE, Basque Foundation for Science, Plaza Euskadi 5, 48009 Bilbao, Spain

## Abstract

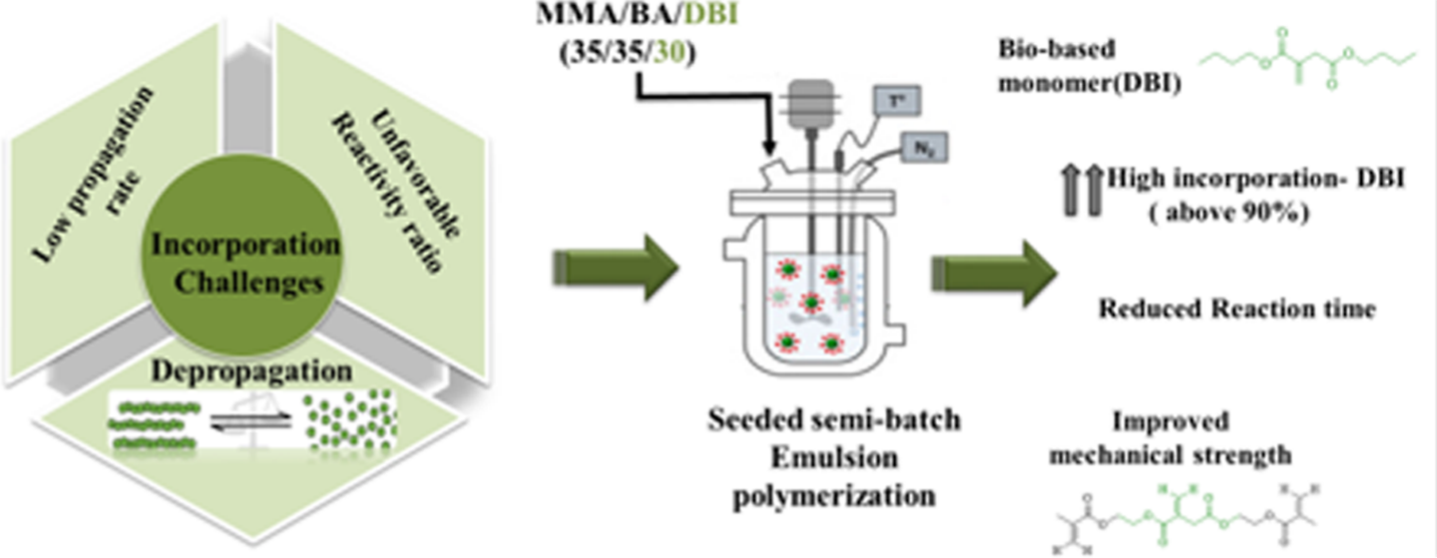

Polymeric derivatives of itaconic acid are gaining interest as biobased alternatives to petroleum-based monomers due to their versatility, renewable nature, commercial availability, and cost-effectiveness. Itaconate ester monomer’s challenges incorporating in (meth)acrylic waterborne polymers are the low propagation rate, unfavorable reactivity ratios, and the depropagation process. To overcome these challenges, the seeded semibatch emulsion polymerization of 100% biobased dibutyl itaconate, methyl methacrylate, and butyl acrylate was investigated at different temperatures. Consequently, 30 wt % DBI was successfully incorporated within waterborne (meth)acrylates in short reaction times (4 h), obtaining high DBI incorporation (>90%). The results demonstrate that DBI incorporation influences the instantaneous monomer conversion, polymer’s microstructure, and mechanical properties. By incorporating a biobased itaconate cross-linker, kinetics and mechanical characteristics of the polymers were improved. This combined approach can be implemented without altering industrial processes, resolving the commercialization dilemma for itaconate monomers to synthesize high-performance biobased polymers for adhesive and coating industries.

## 1 Introduction

The 19th Century Industrial Revolution was a breakthrough in human history, ushering in substantial advances in the production and use of polymers. With the introduction of new technologies based on the widespread use of fossil fuels, synthetic polymers became prevalent and concerns about their environmental impact started to arise.^1^ Emulsion polymerization is one of the major polymerization techniques, giving rise to waterborne polymer dispersions (latexes) which market size is expected to increase for about 6.5% in the period 2024 to 2030.^2^ Nowadays, synthetic latex-based materials are used to make a variety of items, including paints, adhesives, balloons, athletic equipment, gloves, swim caps, tires, and rubber bands. However, crude oil, natural gas, and coal are still the principal raw materials used for their synthesis and the common monomers polymerized in emulsion polymerization are derived from petroleum feedstock.^3,4^ The progressive decline of fossil resources together with the growing instability of petroleum prices, consumer demands, environmental concerns, and stringent regulations on greenhouse gas emissions (particularly CO_2_) has pushed our society to develop sustainable and environmentally acceptable alternatives to replace fossil fuels as raw materials for the production of polymers.^5,6^ This is well illustrated by the vast number of research articles and papers that have reported polymers derived from renewable resources, also referred to as biobased polymers.^7−15^ In this context, programs like Agenda 21 motivate researchers to look for renewable natural resources to guarantee sustainable development in this century.^16^ Biomass is the most viable option since the products are renewable and do not contribute to fossilized carbon release.^17,18^

There are different sources for synthesizing biobased monomers, such as lignin, carbohydrate derivatives, plant oils, and terpenes.^6,19−24^ A huge number of reports were published regarding the synthesis of biobased monomers, polymerized afterwards using different polymerization approaches, including emulsion polymerization which is an environmentally friendly process due to the use of water as the polymerization medium.^25−29^ However, despite years of intense research and development, few investigated systems have proven feasible for industrial-scale polymerization. This is mainly due to the scarcity and unavailability of raw materials, which makes large-scale production challenging.^4^ Therefore, it must be emphasized that the shift from petroleum-sourced polymers to biobased alternatives has been slower than expected from an industrial point of view, as highlighted in a recent study addressing current advances in polymers.^30^

Itaconic acid (IA) is receiving a lot of attention as a green raw material because it is produced industrially by fermenting carbohydrates with filamentous fungi *Aspergillus terreus*, making it one of the most promising building blocks for biobased polymers.^31−33^ Despite structural similarities with methacrylic and acrylic acids, itaconic acid polymerizes much more slowly than these monomers. The extra methyl group at the double bond slows down the total polymerization rate by at least a factor of 10 when shifting from acrylic to methacrylic acid.^34^ Further reduction in the polymerization rate by approximately 2 orders of magnitude occurs upon substitution of the methyl group depending upon the bulkiness of the substituent.^35,36^ The rate constant of each free radical polymerization reaction step is distinctly affected by its substitution.

The main characteristics of the IA include its abundant availability, sustainability of the process, and cost-effectiveness, alongside its ability to generate nonhazardous waste during its production. However, what sets it apart even further is its unique capability to yield 100% biobased itaconate ester monomers by reacting with biobased alcohols.^9,37−41^

Therefore, itaconate esters seem to be ideal candidates to substitute petroleum-based monomers with greener alternatives. However, due to their distinct characteristics, their incorporation in emulsion formulation has been shown to be very challenging. On the one hand, the termination rate coefficient (*k*_t_) and the propagation rate (*k*_p_) of itaconate esters are significantly lower than those of common monomers under similar reaction conditions because the bulky groups surrounding the vinyl functionality hinders the radical propagation.^42−48^ On the other hand, the reactivity ratios with some of the typical monomers used in emulsion polymerizations are still unknown. Furthermore, itaconate ester monomers suffer depropagation, which negatively affects the polymerization process and polymer characteristics.^36,47,49^ For emulsion polymerization, where the temperatures are below 100 °C, depropagation is not significant, even though for some monomer families like methacrylates, these reverse reactions cannot be neglected.^50^ Itaconate esters are even more affected by the depropagation process because of a much lower ceiling temperature of 110 °C.^36^ Even at reaction temperatures below 100 °C, depropagation exerts a significant impact on itaconate radical polymerization kinetics, with a significant reduction in the propagation. It should be also mentioned, that itaconate esters’ microstructure might be also affected by the influence of chain transfer to the solvent or monomer for instance.^36,51^

The addition of itaconate esters as comonomers in radical copolymerization presents an opportunity to increase the renewable content of coatings and adhesive formulations.^52^ Nevertheless, as a consequence of the lower propagation rates and chain transfer reactions, the itaconate ester copolymerization with emulsion monomers has presented multiple challenges, such as long reaction times,^53−55^ low itaconate ester incorporation,^55,56^ and short kinetic chain length of the copolymers.^57^

Even in cases where high conversions were achieved, they were far from the commercial restrictions of residual monomers. In addition, none of the works presents a deeper understanding of the depropagation process, the chain transfer reactions, nor possible solutions. This is why in this work, a polymerization approach to resolve the challenges of long reaction times and low itaconate ester incorporation is presented. For that aim, a seeded semibatch polymerization process has been designed copolymerizing methyl methacrylate (MMA) and butyl acrylate (BA) with dibutyl itaconate (DBI) in which a redox initiator is used. As the radical generation process in redox systems depends only on the initiator’s concentration and not on the reaction temperature, the effect of the reaction temperature in the depropagation process as well as in the chain transfer reactions^58^ could be analyzed, providing understanding on not only the polymerization kinetics but also the microstructure of the final polymer. In the second part of the article, a strategy to improve the low performance of copolymers where the DBI was incorporated is offered based on a novel cross-linker synthesized starting from IA in the formulation.

This work represents a significant advancement in leveraging the excellent characteristics of IA and, more precisely, DBI as a green and abundant raw material. It is a clear strategy to increase the biobased content of waterborne polymer dispersions while also enhancing the properties of the polymers for high-performance coating applications.

## 2 Experimental Section

### 2.1 Materials

**Figure 1:**
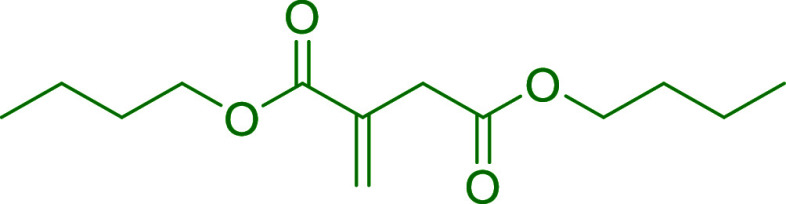
Chemical structure of dibutyl itaconate (DBI).

Itaconic acid (IA, purity, 99%, Sigma-Aldrich), *p*TSA·H_2_O (purity, 98.5%, Sigma-Aldrich), 2-hydroxyethyl methacrylate (HEMA, 99% purity, Sigma-Aldrich), sodium bicarbonate (NaHCO_3_, purity, 99%, Sigma-Aldrich), sodium chloride (NaCl, purity, 99%, Sigma-Aldrich), sodium sulfate (Na_2_SO_4_, purity, 99%, Acros Organics), toluene (purity 99%, Fisher Scientific), and dibutyl itaconate (DBI, 99% purity, Sigma-Aldrich, see Figure 1) were used as received. The monomers methyl methacrylate (MMA, purity 99.9%) and butyl acrylate (BA, purity 99%) were purchased from Quimidroga. The conventional surfactant Dowfax 2A1 (alkyldiphenyloxide disulfonate) was kindly supplied by Dow Chemical (Midland, Michigan, USA). The components of the redox initiator system *tert*-butyl hydroperoxide (TBHP), 70 wt % aqueous solutions, (Luperox from Sigma-Aldrich), and Bruggolite (FF6, Bruggemann) were used as received. Hydroquinone (HQ, purity 99%, Panreac) was employed to stop the reactions. The conventional cross-linkers allyl methacrylate (AMA, purity 99%, Sigma-Aldrich) and ethylene glycol dimethacrylate (EGDMA, purity 98%, Sigma-Aldrich) were used as received. Deuterated DMSO (DMSO-*d*_6_, purity 99.9%, Eurisotop), dimethylformamide (DMF, purity 99%, Sigma-Aldrich) as an external reference in NMR and deuterated chloroform (CDCl_3_-*d*, purity 99.9%, Eurisotop), and tetrahydrofuran (THF, purity 99%, Macron) for GPC measurements were used without further purification, respectively. Distilled water was used in all of the reactions.

### 2.2 Cross-Linker Synthesis Based on Itaconate Ester with a Methacrylic Functionality

**Figure 2:**
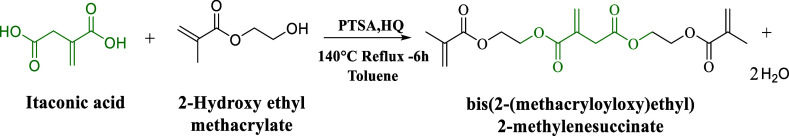
Synthesis of bis[2-(methacryloyloxy)ethyl]2-methylene-succinate (IH).

Bis[2-(methacryloyloxy)ethyl]2-methylene-succinate (IH) was synthesized via esterification of IA and HEMA with a 1:1 mol ratio, as shown in Figure 2. Toluene (150 mL) was poured into a round-bottom flask equipped with a stirrer and a water separator (Dean–Stark apparatus). The reactants, itaconic acid (15 g, 0.11 mol), HEMA (15.01 g, 0.11 mol), and hydroquinone (0.12 g, 0.001 mol) were added and dissolved in the flask. The mixture was heated to 140 °C under stirring and the *p*TSA·H_2_O (2.2 g, 0.01 mol) as a catalyst was dropped by a syringe into the flask slowly, and the esterification started with the reflux of toluene for 6 h. The yellowish solution was quenched with NaHCO_3_ solution (1 M, 100 mL) and then extracted with NaHCO_3_ solution (1 M, 2 × 50 mL) before washing with brine (50 mL). The organic layers were dried over Na_2_SO_4_, filtered, and distilled under reduced pressure using a rotary evaporator to remove the remaining toluene.

### 2.3 Polymerization Process

A two-step seeded semibatch polymerization process was designed. A 500 mL jacketed glass reactor equipped with a reflux condenser, N_2_ inlet, temperature probe, stainless steel agitator, and a sampling tube was used. The reactor was first charged with water, Dowfax 2A1 and MMA/BA mixture stirred at a rate of 200 rpm under an N_2_ atmosphere. When the temperature reached the desired value (75 °C), KPS initiator in an aqueous solution was added as a shot. The system was allowed to react batch-wise for 2 h. The recipe used to synthesize the seed in a batch process is given in Table 1. The solids content (S.C.) of the seed was 20% and this seed was further used in all of the seeded semicontinuous emulsion polymerization reactions.

**Table 1:** Recipe for the Seed (MMA/BA) Synthesized in Batch (20% S.C.)

ingredients		wt (%)	amount (g)
high *T*_g_ monomer	MMA	10	3.75
low *T*_g_ monomer	BA	10	3.75
emulsifier	Dowfax 2A1^a^	2	3.35
initiator	KPS^a^	1	0.75
continuous phase	water	80	29.6

a Weight % based on total monomer content.

In the second step, the growth of the seed was performed in a stirred tank reactor with a 100 mL capacity. The reactions were performed in a jacketed glass reactor fitted with a nitrogen inlet, a thermocouple, a condenser, a feeding inlet, and a stainless steel anchor-type stirrer controlled by an automatic control system (Camile TG, Biotage). The polymerization process included loading the seed in the reactor and purging with N_2_ until the end of the reaction, under stirring at 200 rpm. 37.5 g of seed and water were charged in the reactor, and once the reactor reached the desired temperature, the TBHP was added as a shot. At the same time, the feeding of the pre-emulsion made of water, Dowfax 2A1, FF6, and the monomer mixture, MMA/BA and the DBI, to reach a final 40% content of the itaconate ester monomer, was started as shown in Table 2. The feeding period was carried out for 3 h. Once the feeding was finished, the reaction was left to react batchwise for an additional 1 h to convert the residual monomer present in the system.

**Table 2:** Recipe for the Seeded Semicontinuous Reactions (40% S.C.)

ingredients		wt (%)	amount (g)
high *T*_g_ monomer	MMA	26	10.25
low *T*_g_ monomer	BA	26	10.25
biobased monomer	DBI	30	12
emulsifier	Dowfax 2A1^a^	1	0.7
redox initiator	TBHP^a^	1	0.33
	FF6^a^	1	0.47
cross-linkers	AMA/IH^b^	1	0.87/2.45
continuous phase	water		28.5

a Weight % based on the total monomer content.

b mol % based on the total monomer content.

For the sake of comparison, a reference latex (IE-0) was synthesized with a monomer mixture of MMA/BA (50/50) and five different polymerization reactions were carried out at different temperatures. This temperature range was selected due to the work of Szablan et al.^36^ in which they analyzed the depropagation effect in solution polymerization and concluded that the effective propagation rate coefficient (*k*_peff_) for the dibutyl itaconate monomer was around 65 °C (check the Supporting Information for further details). In this work, the polymerization method is different (emulsion vs solution) as well as the monomer concentration, and therefore, the selected temperature range was varied between 50 and 90 °C. Thus, according to several studies, depropagation’s negative effects on reaction rate are significantly mitigated in copolymerization systems by adding nondepropagating monomers, such as BA and MMA.

Additionally, four different polymerizations were carried out using three different cross-linkers at 75 °C. Two conventional cross-linkers such as AMA, EGDMA, and the biobased cross-linker synthesized following the procedure detailed in Section 2.2. Two latexes were used as reference latexes, which were made of MMA/BA with the cross-linkers, and the other two experiments were made of MMA/BA/DBI along with the cross-linkers. After the synthesis, the obtained latex was filtered through a nylon mesh. The final solids content for the synthesized latex was kept at 40%.

The copolymer composition in terms of both the weight and the molar fraction of the synthesized latexes, together with the nomenclature used in the present paper, is reported in the Supporting Information in Table S1. The first number of the latex name refers to the weight percentage of the biobased monomer, while the second number corresponds to the reaction temperature.

### 2.4 Characterization Methods

#### 2.4.1 Latex Characterization

The solid content of the latex was calculated gravimetrically. It should be mentioned that during the semicontinuous polymerization processes, two different conversions were defined: the instantaneous conversion, which takes into account the monomer fed at each time to the reactor; and the overall conversion, which considers the whole monomer amount that is going to be fed into the reactor. All the details for the conversion calculation can be found in the Supporting Information.

The DBI monomer conversion was measured by ^1^H NMR. The representative NMR spectra and details are given in the Supporting Information (Figure S2).

The amount of coagulum was measured gravimetrically. The latex was filtered with an 80 μm nylon mesh, and the unfiltered matter was dried in an oven until constant weight was achieved.

The *Z*-average particle diameter was measured at 25 °C and 173° backscatter angle by using dynamic light scattering (DLS) Malvern ZetaSizer Nano-S instrument equipped with a 633 nm red laser. Before the analysis, the withdrawn samples were diluted with a Milli-Q water solution to prevent multiple scattering.

The *Z*-average obtained was used to determine the evolution of the number of particles (*N*_p_) during the reactions, following the below equation.
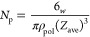
1where *w* is the amount of monomer (g), the density of the polymer is ρ_pol_ (g/mL) using the corresponding for each monomer, and *Z*_ave_ is the diameter obtained from the DLS.

Particle size distributions were obtained using capillary hydrodynamic fractionation in a CHDF3000 instrument (Matec Applied Sciences). The samples were run at a flow rate of 1.4 mL/min at 35 °C with a sample concentration of 1 to 2.5% of S.C. of the latexes. The carrier used to perform the measurement was a 1X GR 500 carrier from Matec.

Gel permeation chromatography (GPC) was used to determine the molar mass distribution (MWD) of the soluble polymer. The polymer was dissolved in pure grade THF in a concentration of 5 mg of polymer/1 mL of THF. This solution was filtered through a 10 μm syringe filter into a GPC vial, a drop of toluene was added as reference, and the sample was measured in the GPC equipment. The GPC setup consisted of a pump (LC-20A, Shimadzu), an autosampler (Waters 717), a differential refractometer (Waters 2410), and three columns in series: Styragel HR2, HR4, and HR6, with pore sizes ranging from 10^2^ to 10^6^ Å were used. Chromatograms were obtained at 35 °C using a THF flow rate of 1 mL/min. The equipment was calibrated using polystyrene standards.

The gel content by definition is the fraction of polymer that is not soluble in a good common solvent, such as THF. The gel fraction was measured by Soxhlet extraction. To measure the gel content, glass fiber square pads (CEM) were used as backing. A few drops of latex were placed on the filter (weight, *W*_1_) and dried at 60 °C overnight. The filter together with the dried polymer was weighed (*W*_2_) and a continuous extraction with THF under reflux in the Soxhlet for 24 h was done. After this period, the wet filter was weighed (*W*_3_) and dried overnight. Finally, the weight of the dry sample was taken (*W*_4_). The gel content was calculated as the ratio between the weight of the insoluble polymer fraction and that of the initial sample, as shown below.
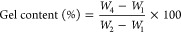
2

#### 2.4.2 Film Characterization

The polymeric films were prepared by casting the latexes in silicone molds for 7 days at 25 °C and 55% relative humidity (RH). For tensile tests, four specimens of each sample were cut with dumbbell shape according to ASTM D 882 (9.53 mm of length and 3.18 × 1 mm of cross-section). The tests were carried out in a universal testing machine TA HD plus texture analyzer equipment (Texture Technologies), at 23 °C and 50% relative humidity, by applying an elongation rate of 25 mm/min, according to ASTM D 638.

The glass transition temperature (*T*_g_) was determined by differential scanning calorimetry (DSC, Q2000, TA Instruments). 3–5 mg of samples were placed in an aluminum hermetic pan. The sample was first heated to 200 °C with a heating rate of 10 °C/min and kept isothermal for 2 min. Then, they were cooled to −50 °C with a cooling rate of 10 °C/min and kept isothermal for 2 min. The second heating run was carried out at 10 °C/min, and this second measurement was used to determine the *T*_g_ of the polymers.

## 3 Results and Discussion

### 3.1 Synthesis of Waterborne Latexes Incorporating Itaconate Ester as a Biobased Monomer into MMA/BA

The seeded semibatch process is widely employed as a copolymerization strategy in emulsion polymerization since it not only provides better control of the reaction heat but also allows tuning the feeding parameter to control the composition of the polymeric chains by overcoming the challenges of unfavorable reactivity ratios. Yet, copolymerization of the itaconate ester monomers comes with certain shortcomings, as mentioned in the Introduction, attributed to a large extent to the equilibrium in monomer concentration caused by depropagation. As this process depends immensely on the reaction temperature, the effect of temperature was studied on the incorporation of DBI into the MMA/BA polymer in a two-step emulsion polymerization process. In the first step, a MMA/BA seed was synthesized batch-wise, obtaining high conversion (99%), no coagulum, and an average particle size of 75 nm. In the second step, the seed was grown by feeding the pre-emulsion mixture (Table 2) at a constant rate for 3 h, followed by 1 h postpolymerization until the final target solid content of 40% was reached. Table 3 summarizes the main properties including conversion of the monomers, the coagulum content, and average particle size (*d*_p_) for the six polymerizations carried out in this work varying the reaction temperature in a range of 50–90 °C. It should be mentioned that the pH of the seed was 1.8 and the pH of the final latex was in the range of 5–6.

**Table 3:** Comparison of the Characteristics of 40% S.C. IE-Based Latexes and the Reference Latex Using Redox Initiator

run	S.C. (%)	MMA/BA Xt (%)	IE conversion (%)	coagulum (%)	*d*_p_ (nm) DLS
IE-0 (REF)	40	100		0	125
IE-DBI_30_-T_50_	37	98	89	3	142
IE-DBI_30_-T_65_	37	96	90	3	145
IE-DBI_30_-T_70_	38	97	92	4	140
IE-DBI_30_-T_75_	39	100	93	2	133
IE-DBI_30_-T_90_	33	84	80	3	132

In all the polymerizations, stable latexes with solids content above 37% were obtained, except for the run carried out at 90 °C, where it was 33%. In all the runs with DBI, a coagulum in a range of 2–4% was observed. The results indicate total conversion of the MMA/BA monomers, as well as high IE conversion of >90% in all the cases except for run IE-DBI_30_-T_90_, which shows conversions of 84 and 80% for MMA/BA and IE, respectively. Even if the optimum *k*_peff_ for dibutyl itaconate monomer was calculated to be 65 °C based on the work of Szablan et al.,^36^ experimentally the run carried out at 75 °C (IE-DBI_30_-T_75_) resulted in the highest conversion and the least quantity of coagulum (2%). This might be related to the different experimental conditions used. For instance, the monomer concentration in the system is different, and this is key when calculating the *k*_peff_.

**Figure 3:**
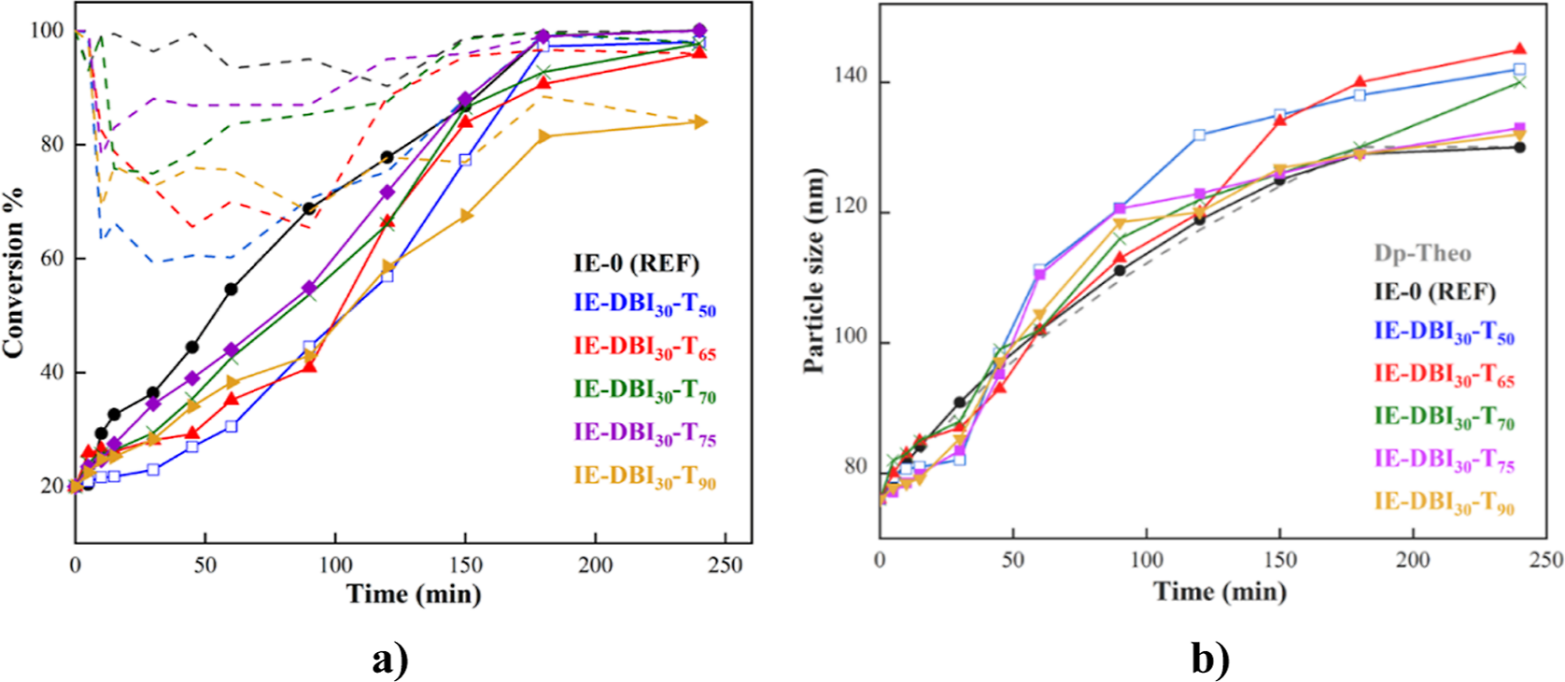
(a) Instantaneous conversion (dash lines) and overall monomer conversions (full lines) of MMA/BA monomers in the seeded semibatch experiments containing IE. (b) Evolution of the intensity–average particle size (full lines) measured by DLS and the theoretical evolution of particle size (dash line).

Figure 3a shows the instantaneous and global conversion of the acrylic monomers for the six polymerizations carried out. It can be seen that the MMA/BA polymerization (IE-0) was performed under starved conditions as the instantaneous conversion was high, above 90% during the whole feeding period. However, when the IE was incorporated into the feeding, a significant effect on the reaction kinetics was observed. The instantaneous conversion decreased mainly during the first 2 h of the feeding, which indicates monomer accumulation in the system, and then it increased steadily, obtaining high conversions at the end of the reaction. In the case of IE-DBI_30_-T_70_ and IE-DBI_30_-T_75_, the instantaneous conversion was high above 80% during the whole process. However, this monomer accumulation was even more pronounced in runs at 50, 65, and 90 °C (IE-DBI_30_-T_50_, IE-DBI_30_-T_65_, and IE-DBI_30_-T_90_), where even at the early stages of the feeding, the instantaneous conversion was lower. The same trend was observed in the DBI instantaneous conversion determined by ^1^H NMR, and the instantaneous conversions of IE-DBI_30_-T_50_ and IE-DBI_30_-T_75_ can be found in Figure S3 of the Supporting Information. The instantaneous conversion of the run carried out at 50 °C, was lower than the one performed at 75 °C within the first 2 h of reaction. However, as the reaction time increased, so did the conversion, reaching, in both cases, high DBI conversions, 89 and 93% respectively. Notably, this inhibition period or monomer accumulation has also been reported by other authors when working with IE.^55,59−61^ Anyway, the DBI conversion increased gradually with the feeding, and despite the accumulation of the monomer in the initial 2 h, almost full conversion was reached after the postpolymerization process except for the reaction at 90 °C (IE-DBI_30_-T_90_). This could be attributed to the depropagation effect, which is more pronounced when reaching temperatures close to the ceiling one (110 °C for the dibutyl itaconate).^36^ It is clear that the kinetics of MMA/BA polymerization differ significantly in the presence and absence of a dityl ester, possibly due to a steric hindrance at the reactive center. In addition, the water solubility of the DBI is much lower than that of the MMA and BA (DBI 0.075 g/L at 20 °C,^62^ MMA 15.3 g/L at 20 °C,^63^ and BA 1.7 g/L at 20 °C^64^), which could also impact up to some extent the incorporation of the DBI. However, high conversions of the (meth)acrylates were achieved as well as high DBI incorporation in only 4 h of reaction time with the monomer combination of MMA/BA/DBI in the ratio of 35/35/30 wt %.

Figure 3b shows the average particle size evolution of the different latexes at different temperatures compared with the theoretical value, obtained by assuming a constant number of particles along the reaction and considering neither new nucleations nor coagulation. It can be seen that the reference latex follows the trend of theoretical evolution. Nevertheless, in the other five reactions where the DBI monomer was incorporated, the experimental values were slightly larger than the theoretical values which might indicate partial particle coagulation in the system. To check this, the samples were run in the CHDF, provided in Figure S4 in the Supporting Information. The distributions of particle sizes in all latexes containing DBI were wider and shifted toward larger sizes with respect to the reference latex, demonstrating partial particle coalescence. This behavior could be attributed to the hydrophobicity of the system since the DBI is more hydrophobic than MMA and BA, and it is well-known, that the parking area for a given emulsifier decreases, increasing the hydrophobicity of the monomer which means that more emulsifier is need to stabilize a particle of the same size.^3^

Polymer microstructure was determined by measuring the gel content (THF insoluble polymer fraction) and the average polymer molar masses of the THF soluble fraction, which are both shown in Table 4. Regarding the gel content, it can be seen that almost all the polymer was soluble in THF, meaning that high cross-linked networks were not created neither in the reference nor when the DBI was incorporated. This is mainly due to lower reactivity of the MMA terminated chains for hydrogen abstraction, the absence of abstractable hydrogens in the MMA units and the fact that MMA radicals terminate predominantly by disproportionation. The reference latex’s values, both the gel content as well as the molar mass are in good agreement with values previously reported in the literature.^65^ The absence of gel in the DBI reaction’s sets could be explained by the high fraction of MMA and DBI in the formulation, due to the absence of labile hydrogen in the MMA and DBI units, together with the fact that MMA and DBI radicals terminate predominantly by disproportionation.^35,66,67^

**Table 4:** Properties of 40% S.C. IE-Based Polymers and the Reference Using a Redox Initiator

run	gel content (%)	*M*_w_ (kDa)	*D̵*	*T*_g_ (°C)
IE-0 (REF)	0	257	4.8	17
IE-DBI_30_-T_50_	0	126	2	13
IE-DBI_30_-T_65_	0	117	3.1	10
IE-DBI_30_-T_70_	0	110	3.4	10
IE-DBI_30_-T_75_	0	85	2.3	8
IE-DBI_30_-T_90_	0	48	1.8	2

On the other hand, it becomes evident that as the reaction temperature increased while keeping the molar ratio of the redox components constant, the molar masses decreased consistently. The molar mass distributions are presented in Figure S5 of the Supporting Information, and it can be seen that the distribution shifted toward lower molar masses with the temperature. This suggest that, as depropagation process is more pronounced at increased temperature, the resulting polymer chains are shorter. However, it is surprising that the molar mass of the polymer produced at 75 °C is consistently lower than that at other temperatures despite the observation that at this temperature, the depropagation process might be less pronounced (Figure 3a). This indicates that there are additional events happening that affect the molar mass during this process. It is already well documented in literature that the growing DBI centered oligoradicals present high affinity of chain transfer to monomer,^47,68^ with a chain transfer-to-monomer rate constant about 2 orders of magnitude higher than that of (meth)acrylates.^35,69^ It seems that the chain transfer to monomer together with the depropagation is responsible for the measured low molar masses. At this stage, the authors are developing a mathematical model to justify this hypothesis.

Glass transition temperature (*T*_g_) values show a similar trend as the molar mass; by increasing the reaction temperature, the *T*_g_ decreases. The plasticizing effect of the residual DBI monomer has been already reported by different authors.^53,61,70,71^ In addition, it can be seen that when the reaction temperature is increased, the *T*_g_ decreases. This effect could be due to two reasons; on the one hand, the lower *T*_g_ was measured for the polymer in which the residual DBI content was higher (80%). On the other hand, as the molar mass of the polymer is also much lower increasing the temperature, these small polymeric chains may act as plasticizers decreasing even more the *T*_g_ of the polymer.

**Figure 4:**
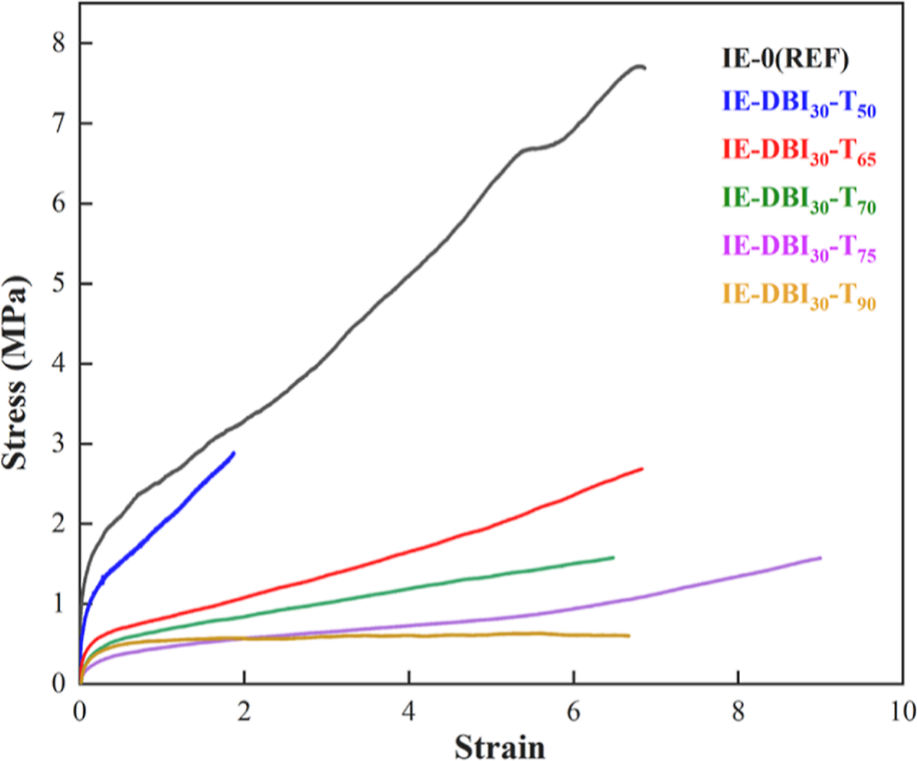
Stress–strain curve of the films containing IE monomer and reference the film using a redox initiator.

The tensile test measurement was used to evaluate the mechanical resistance of the polymer films. Figure 4 shows the stress–strain curves of the films cast under standard atmospheric conditions, whereas the average Young’s modulus, elongation at break, yield stress, and ultimate strength values are shown in Table S2 of the Supporting Information. The films cast with DBI-containing latexes displayed lower tensile strength and higher elongation at break (unless IE-DBI_30_-T_50_) than the reference film. The Young’s modulus of the films containing DBI was in a range of 2–5 MPa, whereas the reference film showed a much higher modulus close to 10 MPa. On the other hand, it should be highlighted that the sample IE-DBI_30_-T_75_ shows a higher elongation at break than the rest of the films containing DBI. As shown in Figure 4 and Table S2, the tensile properties decrease as the reaction temperature increases, in line with the lower molar mass polymers produced at higher temperatures. At first sight, the incorporation of DBI monomer seems to provide a more flexible polymer film since there is a substantial fraction of polymer with low molar mass, which promotes the flow of the polymer chains. In addition, the unreacted DBI may act as plasticizer yielding flexible fiber. During the analysis, all films with itaconate monomer crack at the pace created by the cavitation process and propagate to the interface, resulting in higher elongation of the formed fibrils.

### 3.2 Incorporation of a Biobased Cross-Linker Based on Itaconate Monomer in the MMA/BA/DBI Emulsion Polymers

**Figure 5:**
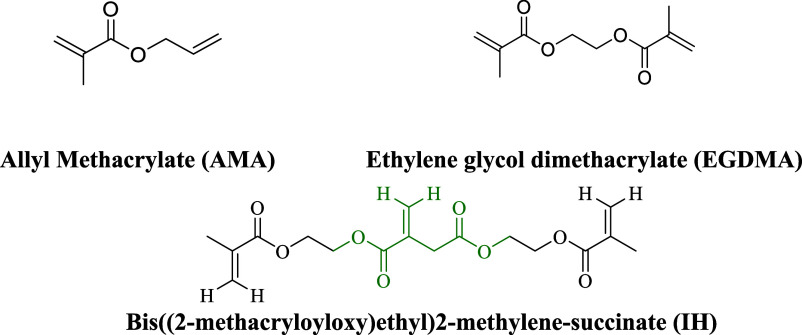
Structure of the conventional (AMA and EGDMA) and biobased (IH) cross-linkers.

In order to improve the mechanical properties of the polymers and to counteract the negative effect of the DBI presence, a novel biobased cross-linker was incorporated into the formulation. The cross-linker was synthesized by an esterification reaction between the IA and HEMA as presented in Figure 2. The resulting product is bis[2-(methacryloyloxy)ethyl]2-methylene-succinate (IH) which has been confirmed by ^1^H NMR, as shown in Figure S1 in the Supporting Information. Apart from the biobased cross-linker IH with a biocarbon content of 30%, two conventional cross-linkers AMA and EGDMA were used for comparison purposes. The chemical structures of the three cross-linkers employed in this study are displayed in Figure 5. The IH is a trifunctional cross-linker with two symmetric methacrylic double bonds and an allylic one, which is more hindered. The main difference between the two conventional cross-linkers is that, while the AMA is an asymmetrical cross-linker, containing a methacrylic and an allylic double bond, EGDMA contains two symmetric methacrylic double bonds. It should be taken into consideration that the reactivity of the different double bonds is likely different, for instance, the methacrylic one would be more reactive than the allylic ones.^72,73^

For comparison purposes and to check the effect that the cross-linker may have not only on the kinetics but also on the microstructure of the polymer, six latexes were synthesized using seeded semibatch emulsion polymerization at a temperature of 75 °C, as at this temperature, the highest instantaneous and DBI conversions were obtained. Three reference latexes without DBI were synthesized, one with each cross-linker, and then the cross-linker was added to the formulation in which the DBI was also incorporated. Targeted solids content (S.C.) was 40%. In all the cases, 1 mol % of cross-linker was used. Table 5 summarizes the main characteristics of the six polymerizations with cross-linkers carried out in this work.

**Table 5:** Comparison of the Main Properties of 40% S.C. IE-Based Latexes and the Reference Latexes Using Redox Initiator along with Crosslinkers

run	S.C. (%)	MMA/BA Xt (%)	IE Conv (%)	coagulum (%)	*d*_p_ (nm) DLS
IE-0-T_75-1%AMA_	37	90		6	146
IE-DBI_30_-T_75-1%AMA_	36	89	70	5	135
IE-0-T_75-1%EG_	40	100		3	180
IE-DBI_30_-T_75-1%EG_	40	99	66	1	149
IE-0-T_75-1%IH_	40	100		2	191
IE-DBI_30_-T_75-1%IH_	40	96	89		158

**Figure 6:**
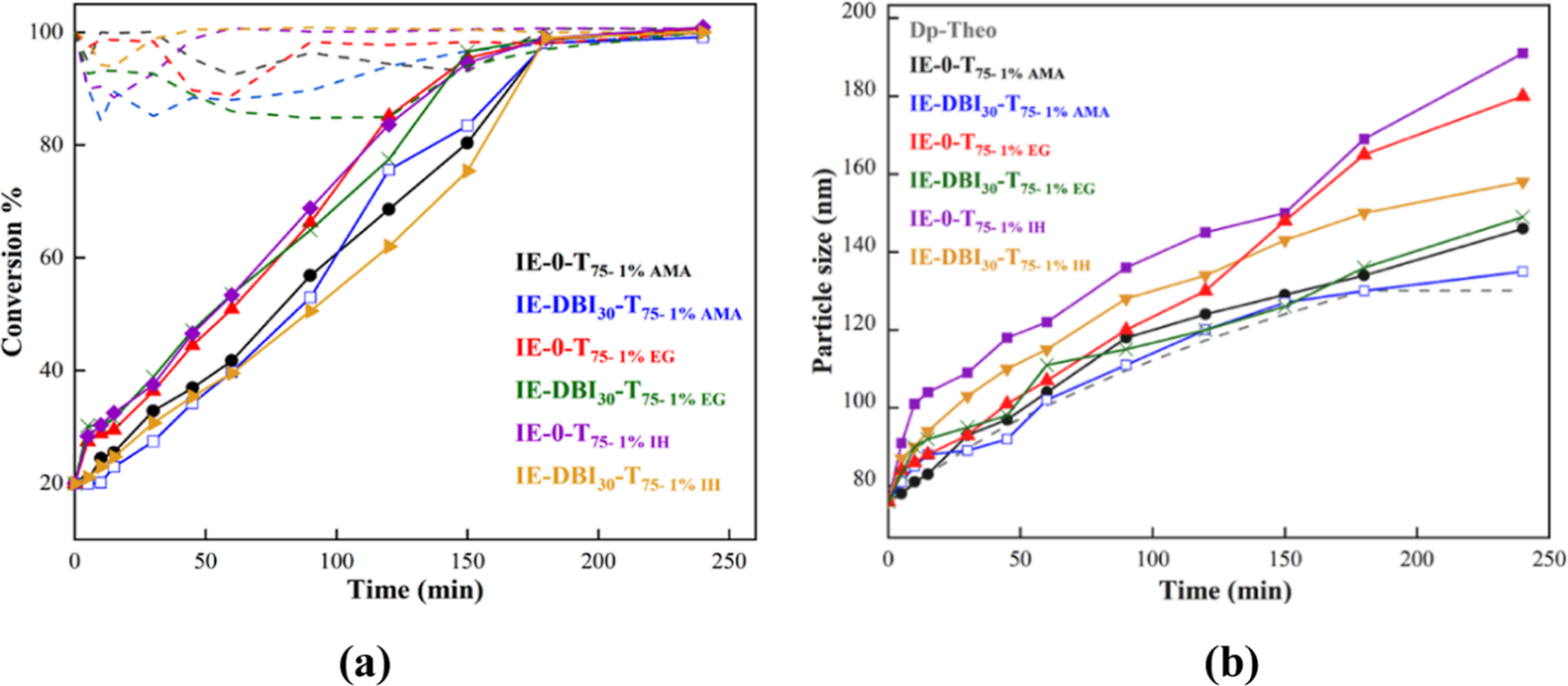
(a) Instantaneous conversion (dash lines) and overall monomer conversions (full lines) of MMA/BA monomers in the seeded semibatch experiments containing DBI and cross-linkers. (b) Evolution of the intensity–average particle size (full lines) measured by DLS and the theoretical evolution of particle size (dash line).

As shown in Table 5 and Figure 6a, high instantaneous (above 80%) and final conversions (above 90%) of MMA/BA were obtained for all of the latexes. Overall, the instantaneous conversions measured for MMA/BA when the DBI was incorporated were higher, which means that the feeding was done under starved conditions and hence, the instantaneous copolymer composition might be very close to that used in the feeding stream producing random copolymer chains. However, a small amount of coagulum was obtained when the cross-linkers were incorporated, which may have affected the final conversion value of MMA/BA measured gravimetrically. It should be mentioned that the coagulum amount was negligible for the latexes synthesized using the biobased IH cross-linker. In addition, not only the final DBI incorporation was the highest when IH was used but also the instantaneous conversion of DBI (Figure S6 in the Supporting Information). The average particle size of the latexes ranged between 135 and 191 nm. Although the amount of coagulum was low, in the evolution of the particle size and comparing the theoretical evolution of the particle sizes (Figure 6b), aggregation between the particles may have happened since the final average size was larger than the predicted one. This effect was more pronounced in the cases where EGDMA and IH were used without DBI.

Regarding the microstructure of the polymers, as expected when adding a cross-linker, all of them presented elevated gel contents, and the higher the gel content, the lower was the measured molar mass of the soluble fraction.

In the three systems, it can be observed that in the presence of DBI, the gel content decreased, likely due to the reduced chain length which might lower the gel content. However, the AMA system presents a higher gel content than the EGDMA and the biobased IH systems. These results are in line with other works published in the literature in which symmetric cross-linkers with more reactive groups leads to lower cross-linked networks.^73^ For instance, in the case of AMA, the methacrylic bonds are more reactive than the allyl bond and would react first. The late consumption of the less reactive allyl bond, which moreover is pendant, will induce the creation of much more cross-linked structures than in the case of the EDGMA and IH cross-linkers, with two methacrylate bonds. In addition, as the IH is a trifunctional cross-linker, the allylic pendant group's reactivity is lower for AMA due to the steric hindrance. Interestingly, the molar mass of the soluble polymer fraction for IE-DBI_30_-T_75_-_1%EG_ and IE-DBI_30_-T_75_-_1%IH_ is very similar to the one obtained in the reaction carried out without cross-linker IE-DBI_30_-T_75_ (Table 4).

The molar masses of the soluble polymer fraction (Table 6) were low due to the incorporation of the larger molar mass chains into the gel. As expected, AMA systems presented the larger gel fraction followed by the IH cross-linker and EGDMA. It should also be mentioned that the molar mass distributions measured for the polymers synthesized using EGDMA and IH were broader than for the polymers synthesized using AMA (see Figure S7).

**Table 6:** Properties of 40% S.C. IE-Based Polymers and the References Using a Redox Initiator

run	gel content (%)	*M*_w_ (kDa)	*D̵*	*T*_g_ (°C)
IE-0-T_75-1%AMA_	84 ± 4	13	1.2	18
IE-DBI_30_-T_75-1%AMA_	55 ± 1	32	1.6	7
IE-0-T_75-1%EG_	52 ± 2	56	1.5	18
IE-DBI_30_-T_75-1%EG_	25 ± 3	82	2.1	6
IE-0-T_75-1%IH_	68 ± 5	62	2.4	19
IE-DBI_30_-T_75-1%IH_	40 ± 2	76	3.1	12

The glass transition temperature (*T*_g_) shown in Table 6 for the three reference polymers adding the cross-linker but without the DBI was very similar. However, the addition of the DBI monomer^74−80^ reduced the *T*_g_ in all systems: AMA (7 °C), EGDMA (6 °C), and biobased IH (12 °C) as in the previous cases (Table 4) where no cross-linker was used in the formulation.

**Figure 7:**
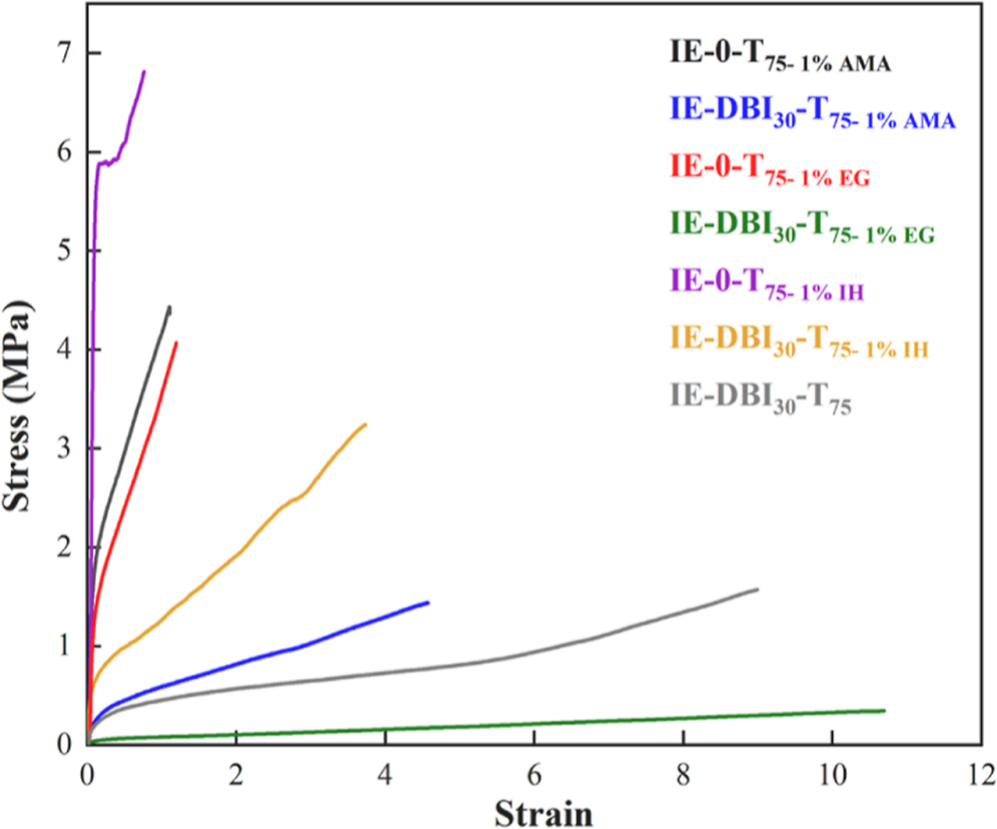
Stress–strain curve of films for IE monomers and reference film using a redox initiator with cross-linkers.

The stress–strain behavior of the polymer films containing the cross-linkers, with and without DBI is shown in Figure 7. The data of the mechanical properties withdrawn from the stress–strain curves are summarized in Table S3 (Supporting Information). It can be seen that for the reference films, with the addition of the cross-linker (Table 6), the gel content was increased, and this improvement was also observed in the mechanical properties of the latexes synthesized using the conventional and partially biobased cross-linkers. However, when the DBI was added to the formulation, the mechanical properties were poorer than in the cross-linked system without DBI, likely due to lower gel content and low *T*_g_.

However, it should be mentioned that the addition of AMA and IH cross-linkers in DBI systems enhanced the mechanical properties with respect to the DBI containing polymer synthesized under the same conditions but without a cross-linker. For instance, Young modulus’ values increased from 1.3 MPa for the IE-DBI_30_-T_75_ system up to 2.5 MPA for the IE-DBI_30_-T_75-1%IH_. This clearly shows that the use of a suitable cross-linker might be one of the solutions for the improvement of the performance of these films.

The biobased IH cross-linker outperformed the AMA cross-linker in terms of tensile strength. This could be attributed to the higher DBI incorporation in the polymer, 70% for the AMA system and 89% for the IH. Higher is the incorporation, less free DBI will be present in the system that acts as a plasticizer. This will have a positive impact on the *T*_g_ and mechanical properties of the polymer, increasing both of them.

## 4 Conclusions

This work has been focused on increasing the biobased content of (meth)acrylic waterborne polymers produced by a two-step emulsion polymerization process, through the incorporation of biobased itaconate ester monomers synthesized from IA, precisely DBI.

When 30% of DBI was incorporated into MMA/BA latex, even if the instantaneous conversion of MMA/BA was decreased initially during the feeding period, a high final conversion of them was achieved at the end of the process. Additionally, the incorporation of the DBI was high during the whole process, obtaining high incorporation values (>90%). The reactions performed at different temperatures in the range of 50–90 °C, showed that increasing the reaction temperature, the molar mass of the system was decreased, likely owing to the depropagation and transfer to monomer events. This decrease had a direct impact on the mechanical properties of the polymer. Moreover, the free DBI present in the system may act as a plasticizer, decreasing the *T*_g_ of the final polymer as well as the mechanical properties.

To mitigate the effect of low molar masses on the mechanical strength of DBI containing polymers, the reactions were performed by including three different cross-linkers in the formulations. Two conventional cross-linkers were used, an asymmetric one AMA and a symmetric one EGDMA together with a novel but partially biobased cross-linker IH. The presence of a cross-linker, on the one hand, improved the conversion and DBI incorporation, and on the other hand, it resulted in cross-linked structures with enhanced mechanical properties, being this effect more pronounced when the partially biobased cross-linker was used.

In a nutshell, the study reveals that despite initial concerns about instantaneous conversion and polymer microstructure due to the unknown reactivities, the IE monomer depropagation and monomer transfer reactions, 30 wt % DBI was successfully incorporated, considerably reducing the polymerization time to a 4 h reaction time, with improved kinetics and mechanical properties when using a novel and partially biobased cross-linker. This study opens the possibility to start thinking on the commercialization of coatings and adhesive containing DBI even though improvement during the postpolymerization may be necessary to increase the final incorporation of the itaconate monomers.

## Supporting Information Available

Equilibrium state related to the depropagation mechanism; latex characterization (solids content of the latex); ^1^H NMR spectra in DMSO-*d*_6_ of bis[2-(methacryloyloxy)ethyl]2-methylene-succinate; summary of the itaconate ester-based latex reactions and its characteristics; ^1^H NMR spectra in CDCl_3_ of IE monomer latex at different reaction times; instantaneous conversion of IE monomer using redox initiator and with a cross-linker; particle size distribution measured by CHDF using a redox initiator; molar mass distribution of latexes with IE monomers and reference using redox initiator and with cross-linker; and mechanical properties of the latexes with IE monomers and reference using a redox initiator and with a cross-linker (PDF)
