# Supplementary material for: Overcoming Challenges of Incorporation of Biobased Dibutyl Itaconate in (Meth)acrylic Waterborne Polymers
Source: Biomacromolecules. 2024 Jul 31;25(8):5310–20. doi: 10.1021/acs.biomac.4c00739 (PMC11323018; doi:10.1021/acs.biomac.4c00739)
Supplement: Supplementary file 1 — bm4c00739_si_001.pdf [file bm4c00739_si_001.pdf]

Supporting Information Overcoming challenges of incorporation of bio-based itaconate esters in (meth)acrylic waterborne polymers

Jyoti Gupta[1], Radmila Tomovska[1,2], Miren Aguirre[1]

[1]POLYMAT, Kimika Aplikatua Saila, Kimika Fakultatea, University of the Basque Country UPV-EHU, Joxe Mari Korta Zentroa, Tolosa Hiribidea 72, 20018 DonostiaSan Sebastián, Spain.

[2]IKERBASQUE, Basque Foundation for Science, Plaza Euskadi 5, 48009, Bilbao, Spain

The equilibrium state related to depropagation mechanism can be characterized by the extent of the free energy ΔG, existing between the polymer and the monomer.[1]

$$\Delta G = \Delta H - T\Delta S \qquad (1)$$

The polymerization entropies are negative, indicating a loss of degrees of freedom when the monomer becomes part of the chain. Polymerization entropies typically range from -100 to -120 J·K/mol-1. Thus, the two terms on the right-hand side of Eq. (1) are antagonistic. Polymerization progresses under the condition of a negative ΔG. However, in cases where both the entropy and enthalpy of the polymerization reaction are negative, a critical temperature known as the ceiling temperature (Tc) is established. Above Tc, the equilibrium shifts, favoring the reverse propagation (depropagation) reaction, impeding further polymerization. Consequently, the propagation step should be expressed as an equilibrium equation[2–5].

$$P_n + M \underset{k_{dp}}{\overset{k_p}{\rightleftarrows}} P_{n+1} \qquad (2)$$

The rate coefficients for propagation (k_p) and depropagation (k_{dp}) refer to the forward and reverse reactions, respectively. P_n refers to a developing radical chain with a length 'n,' while M represents the monomer involved in the process. The change in enthalpy (ΔH) and entropy (ΔS) is calculated as follows:

$$\Delta H = E_p - E_{dp} \tag{3}$$

$$\Delta S = R\ln\left({}^{A_p}\!/_{A_{dp}}\right) + R ln\,[M] \tag{4}$$

The activation energies and frequency factors of the forward and reverse rate coefficients, represented in the standard Arrhenius form, are E and A, respectively.

$$k_p = A_p \exp\left({}^{-E_p}\!/_{RT}\right) \tag{5}$$

$$k_{dp} = A_{dp} \exp\left({}^{-E_{dp}}\!/_{RT}\right) \tag{6}$$

and the effective propagation rate, denoted by k_p^{eff}, is given by:

$$k_p^{eff} = \frac{k_p - k_{dp}}{[M]} \tag{7}$$

where the k_p^{eff} units are L/mol·s and the [M] mol/L. The exothermic, exentropic nature of most free-radical polymerization dictates that for a given monomer concentration there exists a ceiling temperature above which chain growth will not proceed. Not only does the effective polymerization rate dramatically decrease when this temperature is approached but so does the polymer molar mass since transfer and other side reactions continue at unabated rates.

Latex characterization

The solids content of the latex was calculated gravimetrically by weighting the latex before and after drying as shown in **Eq. (1)**. Latex samples were withdrawn from the reactor at defined intervals. One portion of the sample was weighted in an aluminum cup for conversion calculation. To quench the reaction, 1-2 drops of an aqueous solution of HQ (1 wt% in water) were added to the samples. The aliquots were kept for 2-3 hours under a fume hood and then dried in an oven at 65 °C overnight.

$$S.C. = \frac{\text{Weight of the dry polymer}}{\text{weight of latex}}$$

(~~1~~8)

The monomer conversion was then calculated from the S.C. (solids content) of the latex according to **Eq. (2)**, where NPS are the non-polymeric solids of the sample (initiator,

surfactant, IE, and HQ), m_{latex} is the weight of the latex and m_{mon} is the amount of monomer. During the semi-continuous polymerization processes, two different conversions were defined: the instantaneous conversion, which takes into account the monomer fed at each time to the reactor; and the overall conversion, which considers the whole monomer amount that is going to be fed into the reactor. Both definitions are utilized in this work and the type of monomer conversion used (instantaneous vs overall) will be specified in each case.

$$X_M = \frac{\mathrm{S.C.} * m_{latex-NPS}}{M_{mon}} \qquad (29)$$

Synthesis of itaconate esters with methacrylic functionality

When performed conventionally, the esterification product was analyzed by ^{1}H-NMR and the product obtained was a diester, precisely (Bis(2-(methacryloyloxy)ethyl) 2-ethylene succinate). In **Figure S1**, the assignation of each proton can be seen. The signal at 1.8 ppm corresponds to the (6H) of the methyl groups. The signals at 3.4 and 4.4 ppm to (2H) and (8H) of the methylene groups, respectively, and the signals between 5.6 and 6.3 ppm belong to the 3 different vinyl groups of the diester.

In addition, it can be mentioned that the yield of this reaction was 29%. This yield is low, most probably due to the purification method, during which some product quantity was lost. Regarding the purification, the yellowish solution was quenched with $NaHCO_3$ solution (1M, 100 mL) and then extracted with a $NaHCO_3$ solution (1M, 2 x 50 mL) before washing with brine (50 mL). The organic layers were dried over Na_2SO_4, filtered and distilled under reduced pressure using a rotary evaporator to remove the remaining toluene. During the workup, the unreacted itaconic acid might be washed away.

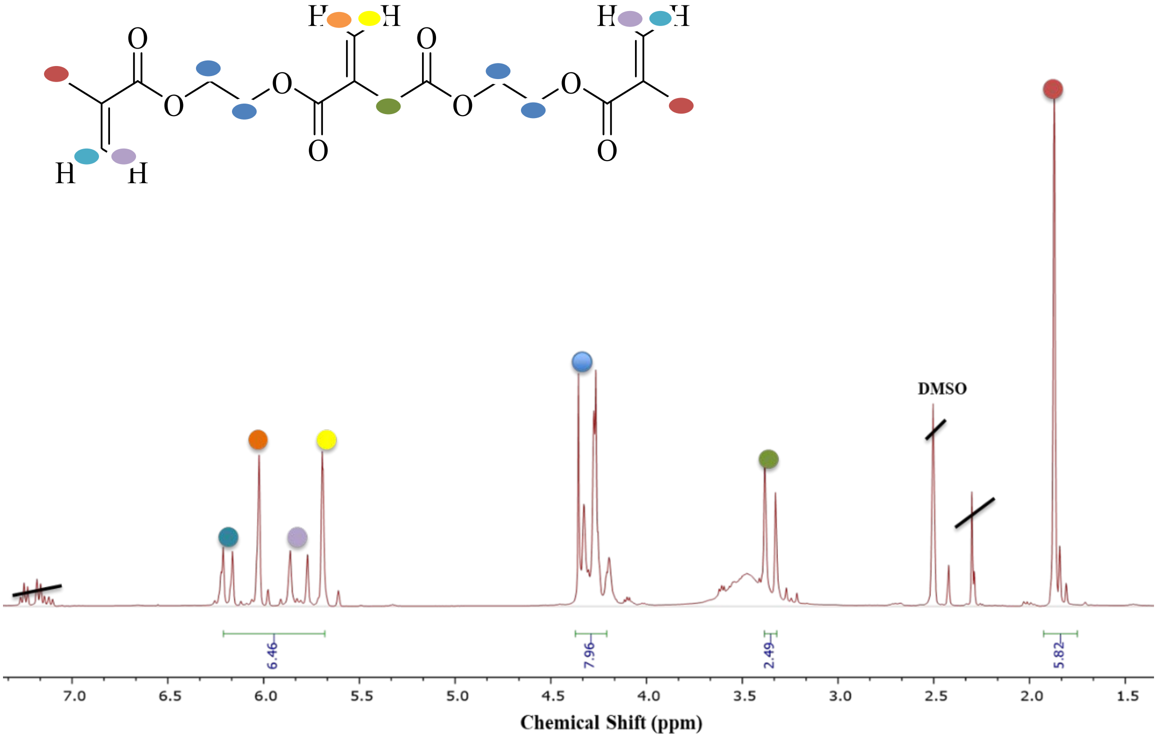

Figure S1.^{1}H-NMR spectra in DMSO-d_6 of bis(2-(methacryloyloxy)ethyl)2-methylene-succinate (IH).

Table S1. Summary of the Itaconate ester-based latex reactions and its characteristics.

Runs	SC (%)	Temperature (℃)	IE content (%)	Crosslinker (mol %)
IE-0 (REF)	40	75	-	-
IE-DBI$_{30}$-T$_{50}$	40	50	30	-
IE-DBI$_{30}$-T$_{65}$	40	65	30	-
IE-DBI$_{30}$-T$_{70}$	40	70	30	-
IE-DBI$_{30}$-T$_{75}$	40	75	30	-
IE-DBI$_{30}$-T$_{90}$	40	90	30	-
IE-0-T$_{75\text{-}1\%\ AMA}$	40	75	-	1
IE-DBI$_{30}$-T$_{75\text{-}1\%\ AMA}$	40	75	30	1
IE-0-T$_{75\text{-}1\%\ EG}$	40	75	-	1
IE-DBI$_{30}$-T$_{75\text{-}1\%\ EG}$	40	75	30	1
IE-0-T$_{75\text{-}1\%\ IH}$	40	75	-	1
IE-DBI$_{30}$-T$_{75\text{-}1\%\ IH}$	40	75	30	1

The IE monomer conversion was measured by ^{1}H-NMR **(Figure S2)**. The conversion of IE monomer in defined intervals of polymerizations was determined from the integration of the monomer peaks at 5.68 and 6.30 ppm (vinyl peak) against the integration of the reference DMF peak at 8.02 ppm. The conversion is calculated by a simple mass balance. NMR samples were prepared by taking 2 mg/mL for ^{1}H NMR in DMSO-d$_6$ and the conversion of IE in the latex was determined at defined intervals of 5, 90, and 270 min. For that, 20 mg of dry polymer were dissolved in 500 µL $CDCl_3$ with 2 µL of DMF as a ~~reference~~ internal standard and were recorded in a Bruker Avance-400 instrument. The same procedure was followed either the crosslinker was used or not.

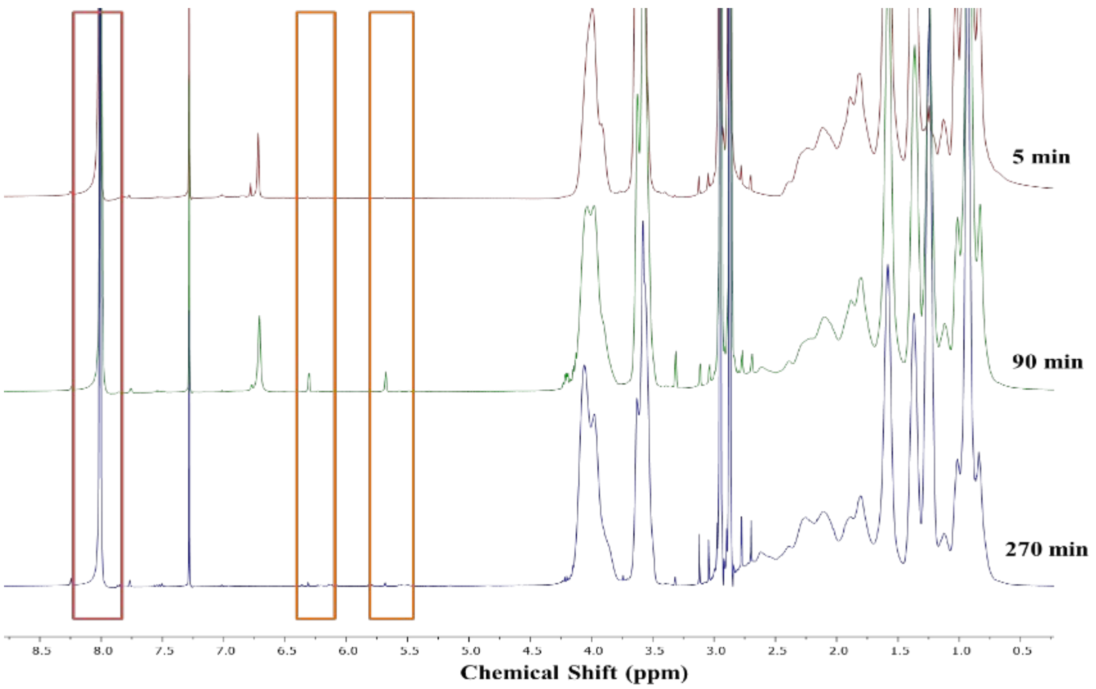

Figure S2. ^{1}H-NMR spectra in $CDCl_3$ of IE monomer latex at different reaction times. The vinyl peaks of IE decrease in intensity as the reaction proceeds (up to the bottom).

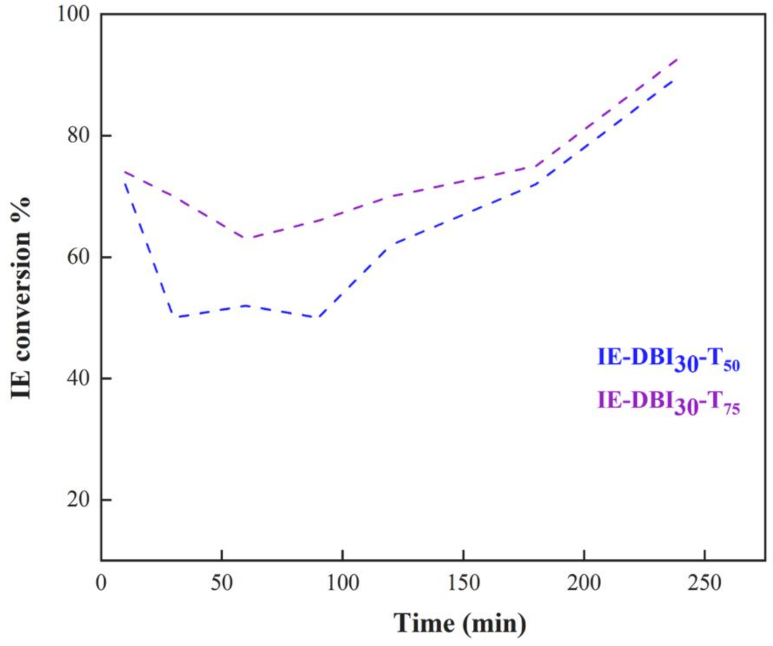

Figure S3. Instantaneous conversion (dash lines) of IE monomer in the seeded semi-batch experiments.

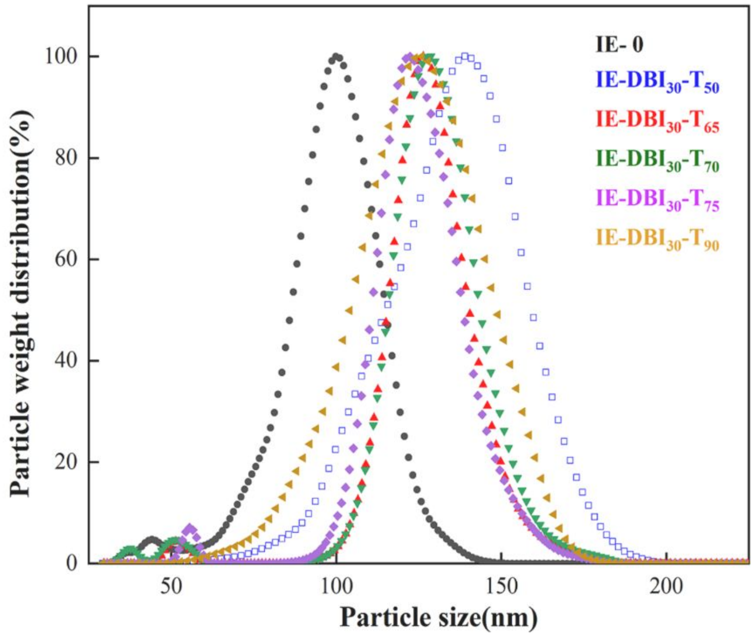

Figure S4. Particle size distribution measured by CHDF.

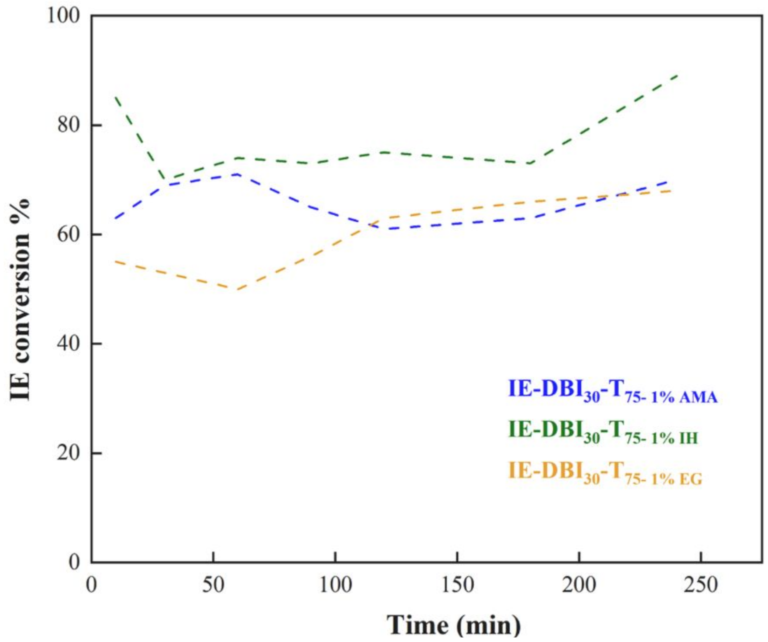

Figure S6. Instantaneous conversion (dash lines) of IE monomer in the seeded semi-batch experiments containing crosslinkers.

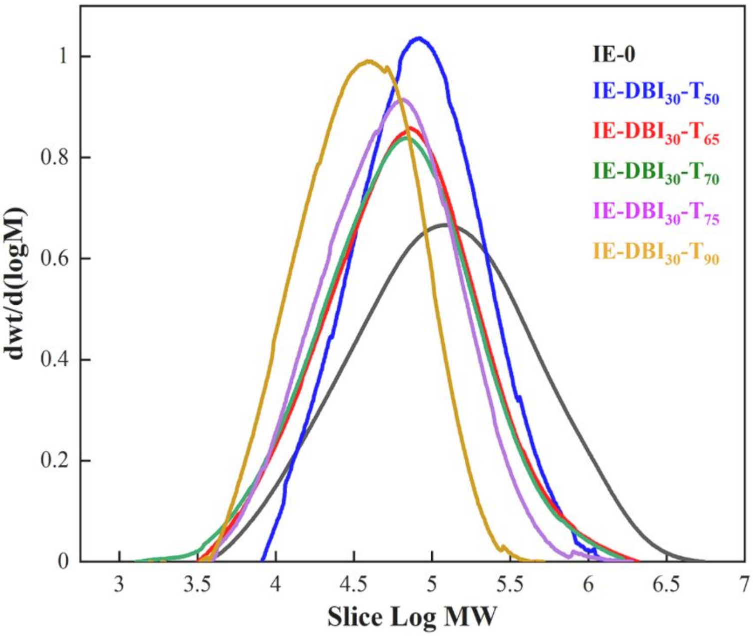

Figure S5. Molar mass distribution of latexes with IE monomers and reference using redox initiator

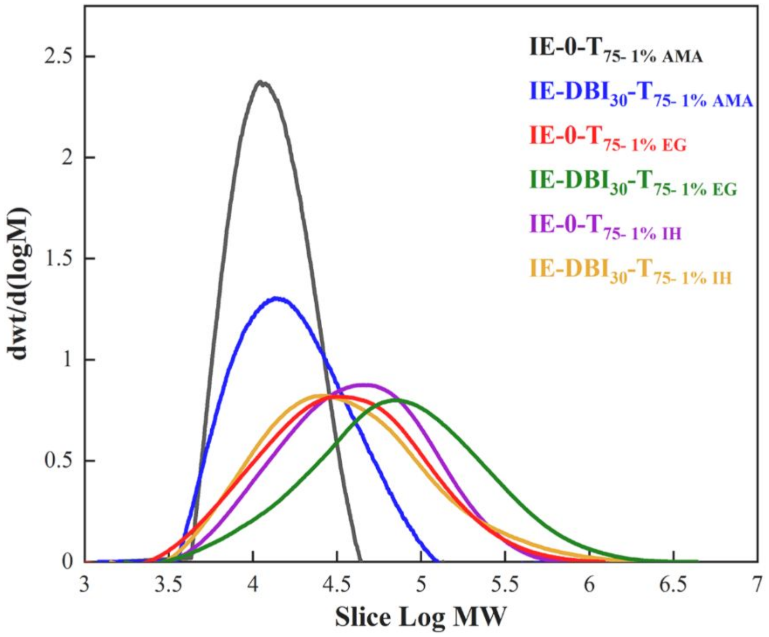

Figure S7. Molar mass distribution of latexes with IE monomers and reference using redox initiator with crosslinkers.

Table S2. Mechanical properties of the latexes with IE monomers and Reference using Redox initiator.

Run	Young´s Modulus (MPa)	Yield stress (MPa)	Elongation at Break (%/100)	Ultimate strength (MPa)
**IE-0 (REF)**	9.6 ± 0.8	2.8 ± 0.9	6.3 ± 0.9	7.9 ± 2.5
**IE-DBI$_{30}$-T$_{50}$**	5.0 ± 3.4	1.4 ± 0.4	2.4 ± 0.8	3.8 ± 1.4
**IE-DBI$_{30}$-T$_{65}$**	2.0 ± 1.8	0.5 ± 0.2	6.6 ± 1.3	3.5 ± 1.0
**IE-DBI$_{30}$-T$_{70}$**	1.9 ± 3.5	0.2 ± 0.1	6.4 ± 2	1.5 ± 0.8
**IE-DBI$_{30}$-T$_{75}$**	1.3 ± 2	0.6 ± 0.1	9.0 ± 2.5	1.6 ± 2.2
**IE-DBI$_{30}$-T$_{90}$**	1.5 ± 0.8	0.2 ± 0.1	6.5 ± 1.3	0.4 ± 0.2

Table S3. Mechanical properties of the latexes with IE monomers and Reference using Redox initiator along with the crosslinkers.

Run	Young´s Modulus (Mpa)	Yield stress (MPa)	Elongation at Break (%/100)	Ultimate strength (MPa)
**IE-0-T_{75}-1% $_{AMA}$**	8.2 ± 2.1	1.8 ± 0.4	1.1 ± 0.2	4.4 ± 0.4
**IE-DBI_{30}-T_{75}-1% AMA**	1.5 ± 0.5	0.3 ± 0.1	4.6 ± 0.3	1.5 ± 0.3
**IE-0-T_{75}-1% $_{EG}$**	7.4 ± 1.1	1.6 ± 1.4	1.2 ± 1.0	4.0 ± 1.0
**IE-DBI_{30}-T_{75}-1% EG**	1.1 ± 1.5	0.1 ± 0.5	10.8 ± 2.0	1.8 ± 0.5
**IE-0-T_{75}-1% $_{IH}$**	33 ± 1.1	5.8 ± 2.1	0.7 ± 1.7	7.8 ± 1.0
**IE-DBI_{30}-T_{75}-1% $_{IH}$**	2.5 ± 1.5	0.5 ± 1.0	3.7 ± 2.0	3.2 ± 0.5

Reference

1. Jones GR, Wang HS, Parkatzidis K, Whitfield R, Truong NP, Anastasaki A. Reversed Controlled Polymerization (RCP): Depolymerization from Well-Defined Polymers to Monomers. *J Am Chem Soc*. 2023;145(18):9898-9915. doi:10.1021/jacs.3c00589

2. Hutchinson RA, Paquet DA, Beuermann S, McMinn JH. Investigation of methacrylate free-radical depropagation kinetics by pulsed-laser polymerization. *Ind Eng Chem Res*. 1998;37(9):3567-3574. doi:10.1021/ie980167p

3. Wang W, Hutchinson RA, Grady MC. Study of butyl methacrylate depropagation behavior using batch experiments in combination with modeling. *Ind Eng Chem Res*. 2009;48(10):4810-4816. doi:10.1021/ie900060x

4. Palmer DE, Mcmanus NT, Penlidis A. Copolymerization with depropagation: A study of α-methyl styrene/methyl methacrylate in solution at elevated temperatures. *J Polym Sci Part A Polym Chem*. 2001;39(10):1753-1763. doi:10.1002/pola.1153

5. Szablan Z, Stenzel MH, Davis TP, Barner L, Barner-Kowollik C. Depropagation kinetics of sterically demanding monomers: A pulsed laser size exclusion chromatography study. *Macromolecules*. 2005;38(14):5944-5954.

doi:10.1021/ma050444l
